# Synthesis and Characterization of Thermally Stable Lignosulfonamides

**DOI:** 10.3390/molecules27217231

**Published:** 2022-10-25

**Authors:** Karolina Komisarz, Tomasz M. Majka, Monika Kurczab, Krzysztof Pielichowski

**Affiliations:** Department of Chemistry and Technology of Polymers, Faculty of Chemical Engineering and Technology, Cracow University of Technology, ul. Warszawska 24, 31-155 Kraków, Poland

**Keywords:** biocomposites, biopolymers, chemical modification, lignosulfonate, sulfonamides

## Abstract

Lignin, a highly aromatic macromolecule building plant cells, and cellulose are two of the most commonly occurring natural polymers. Lignosulfonate is a grade of technical lignin, obtained as a by-product in the paper and wood pulping industries, a result of the used lignin isolation method, i.e., sulfite process. In this work, sodium lignosulfonate is used as a starting material to manufacture sulfonamide derivatives of lignin in a two-step modification procedure. Since this direction of the lignin modification is rather rarely investigated and discussed, it makes a good starting point to expand the state of knowledge and explore the properties of lignosulfonamides. Materials obtained after modification underwent characterization by FTIR, SS-NMR, WAXD, SEM, and TGA. Spectroscopic measurements confirmed the incorporation of dihexylamine into the lignin structure and the formation of lignosulfonamide. The crystalline structure of the material was not affected by the modification procedure, as evidenced by the WAXD, with only minute morphological changes of the surface visible on the SEM imaging. The obtained materials were characterized by improved parameters of thermal stability in relation to the raw material. As-prepared sulfonamide lignin derivatives with a potential application as a filler in biopolymeric composites may become a new class of functional, value-added, sustainable additives.

## 1. Introduction

Lignin is an abundant, natural, highly branched macromolecule with a high ratio of aromatic rings, present in the cell walls of plants alongside cellulose. The structure of lignin is based on the combination of the three building blocks, phenylpropane derivatives known as monolignols: p-coumaryl, coniferyl, and sinapyl alcohols [1,2]. The ratio of each structure is closely connected to the plant source and directly influences the structure and properties of the isolated lignin [3,4]. Difficulties with obtaining lignin with repeatable characteristics hinder the potential application prospects [5]. Currently, only 5% of the produced lignin is used in low-value applications, and the rest is used as a resource for the production of electricity and recovery of energy [6,7]. However, in recent years, the interest in lignin has been resurfacing, mainly because of the highly aromatic character of the macromolecule, which can become a renewable source of aromatic hydrocarbons and an alternative to petroleum-based chemicals [8,9,10,11]. The potential of lignin as a renewable, natural, abundant source of aromatic hydrocarbons was recognized already in the 1950s, but at the same time, almost all of it got wasted away due to the difficulties in obtaining chemically pure lignin. Some of the fungi enzymes, so-called ‘brown rot’, may be used to obtain chemically pure lignin via a time-consuming process, whereas other industrial methods of lignin isolation, such as sulfite, kraft, and soda-pulping processes cause partial destruction of the lignin structure; moreover, their products contain functional groups or impurities originating from the used chemicals [12].

Nowadays, there are four methods used industrially to isolate lignin from lignocellulosic biomass: soda pulping and organosolv, which do not involve sulfur-containing reagents [13], and sulfur-based processes, kraft and sulfite, which yield technical lignins obtained as a by-product of paper and wood pulping industries. Lignosulfonates are the product of the sulfite process [14] in which lignocellulosic biomass is boiled with the aqueous solution of sulfur dioxide. After the addition of the hydroxide of choice (ammonium, calcium, magnesium, or sodium) [6], the corresponding sulfites are formed, and in the subsequent step, sulfite anions present in the solution bind to the lignin structure, creating benzyl sulfonic acid units [14]. Due to the presence of polar sulfonic groups, lignosulfonate is water soluble, and the sulfur content in the lignosulfonate is usually between 4 and 8% [15]. The chemical reactivity of the obtained lignosulfonate varies in accordance with the used hydroxide—ammonium lignosulfonate is the most active, whereas lignosulfonates obtained using calcium hydroxide are characterized by the least chemical reactivity [16]. Lignosulfonates have found their application in catalysis [17,18], as surfactants in oil recovery [19], in blends with chitosan to form coatings [20], and in the manufacturing of spherical micelles [21].

Unmodified lignin exhibits many valuable features, such as biodegradability, good thermal stability, anti-oxidant and anti-microbial properties [22], and UV-ray absorbance [23]; employing various methods of chemical modification allows for further enhancement of the inherent lignin properties. Lignin may undergo esterification [24], etherification [25], epoxidation [25,26], amination [27,28] and other reactions in accordance to the desired outcome—whether obtaining sorbents for the selective absorption of dyes [29,30,31,32], antibiotics [33] or heavy metals [32,34,35,36] from wastewater or dyestuff effluents, manufacturing of additives for epoxy resins [37,38], polyurethane systems [37,39,40,41], wood adhesives [42,43], additive for asphalt binders [44], lubricants [45], surfactants [46], carbon fibers [47,48] or even sunscreens [49]. The presently explored directions of development of the applicability of technical lignins, whether in the form of hydrogels or other forms, mainly focus on four fields of potential utilization: biomedical, agriculture, environment, and electronics [50]. This year only, researchers have described the application of lignosulfonates and other technical lignins to obtain high-efficien absorbents for the removal of Au(III) [51], Cr(VI) [52], Cu(II) [53], Co(II) [54], or Pb(II) [55] ions, amine-functionalized lignins [56] and magnetic lignosulfonate-based adsorbents for the selective removal of dyes [57] and other wastewater contaminants [58], just to name a few. Recent studies are not limited only to sorbents—lignin is a versatile starting material, which can be used to create hydrogels for capacitor and sensor applications [59,60], efficient photoinitiators [61], or can become a cross-linker for bioprosthetic materials of heart valves [62]. The biomedical field also encompasses drug delivery systems, such as pH-responsive systems based on lignosulfonates [63]. Lignosulfonates show great promise as corrosion inhibitors as well [64]. They are also a point of interest in material engineering, for example, in hybrid systems with CaCO_3_, lignosulfonates act as a base for a filler for isotactic polypropylene (PP), positively influencing the thermal stability of the final composites [65], or, after modification of lignosulfonates with nickel(II) hydroxide, the obtained filler exhibits flame retardant properties, improving smoke suppression, promoting char formation and reducing heat release rate of PP composites [66]. Colloidal spheres based on lignosulfonate and cetyltrimethylammonium bromide display excellent UV-absorption properties and good miscibility with high-density polyethylene, improving the mechanical properties, flowability, processability, and reducing the viscosity of the composites [67]. The incorporation of lignosulfonates into the phenol-formaldehyde (PF) foam systems allows for obtaining foams with advantageous thermal insulation properties [68]. Intrinsic UV-blocking and absorption properties of lignin were explored in research on curcumin-encapsulated chitosan/lignosulfonate micelles [69] and demethylated lignosulfonate microcapsules [70], which can be potentially used to impart antioxidant features to food packaging or act as active ingredients in beauty products.

Such a wide array of possible applications researched in recent years shows the importance, versatility, and high potential of lignin as a source of many functional materials which may be a strong alternative for the commercial products used in the contemporary world.

In this work, we present another method of lignin modification, a continuation of our previous research [71]. In the proposed two-step method, the sulfonyl groups present in the sodium lignosulfonate structure undergo a reaction with two different chlorinating agents, thionyl chloride and phosphorus pentachloride, to obtain lignosulfonyl chloride, which is subsequently reacted with a secondary amine. Aside from the work of our group, a similar approach was proposed in a few patents. Sulfonated lignin is reacted with an acid halide, and the formed intermediate product undergoes a reaction with primary, secondary, or tertiary amine [72]. Similar work describes the manufacturing of surfactants for enhanced oil recovery, based on the lignin sulfonamide. In this approach, spent sulfite liquor, lignosulfonate, or kraft lignin was reacted with a primary amine, containing 16 to 22 carbon atoms [73]. Lignin amides and other nitrogen-based derivatives of lignin were obtained by the oxidation of lignin and subsequent reaction of the newly-formed carboxyl groups with aryl or alkyl primary amines [74]. In the patents US4786720 and US4859362, the first step of modification also consists of the incorporation of carboxylic groups into lignin, whether by oxidation, reaction with maleic or fumaric anhydride via the Diels–Alder mechanism or by the reaction of phenolic hydroxyls with chloroacetic acid under alkaline conditions. The carboxylic groups introduced to lignin can react with polyalkeneamines under non-aqueous conditions and yield amidoamines or imidazolines, which can be used as surfactants for tertiary oil recovery or drilling muds [75,76].

Low-molecular sulfonamides are known for their anti-microbial and bacteriostatic properties, and they are still applied for therapeutic uses. The introduction of the sulfonamide groups to the lignin may have an enhancing effect on the inherent anti-microbial properties of lignin, but it can also affect other characteristics of the material, such as thermal stability.

## 2. Results and Discussion

### 2.1. Routes of Lignosulfonate Chemical Modification

**Figure 1:**
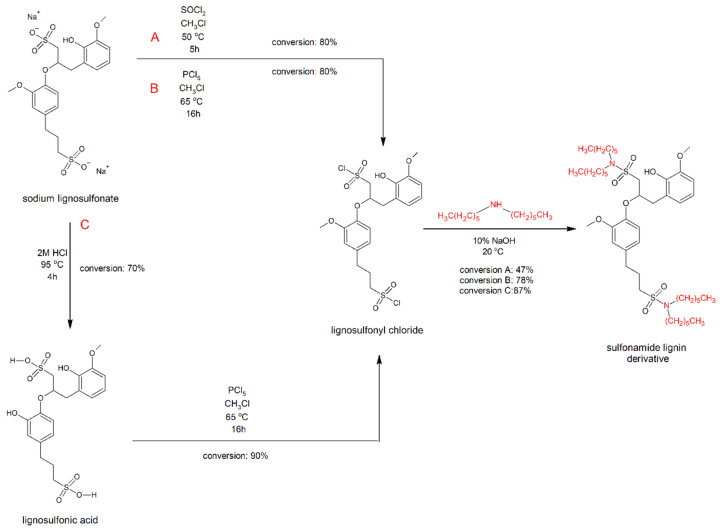
Schematic presentation of the synthetic routes leading to lignosulfonamides.

Sodium lignosulfonate (ligS) was subjected to three methods of chemical modification to yield a sulfonamide derivative of lignin. In the first of the employed methods (route A on the schematic presented in Figure 1), thionyl chloride (SOCl_2_) was used as a chlorinating agent, whereas in routes B and C, the chosen chlorinating agent was phosphorus pentachloride (PCl_5_), with route C having an additional acidolysis step.

All of the materials used and obtained during the investigation are labeled for convenience with acronyms, which are presented in Table 1.

The synthetic routes are described in greater detail in the Section 3.

### 2.2. Fourier Transform Infrared (FTIR) Spectroscopy

**Figure 2:**
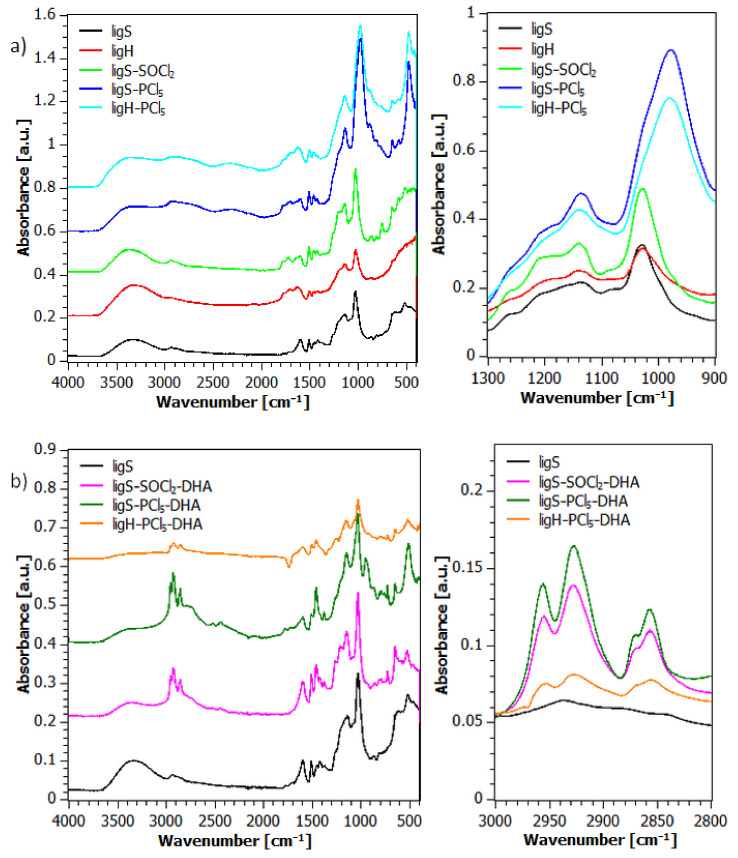
FTIR spectra of the materials obtained after the first (**a**) and the second (**b**) step of the modification procedure.

Infrared spectra obtained for the investigated samples are presented in Figure 2.

The spectrum of the unmodified lignosulfonate (ligS) consists of the bands characteristic of the lignin derivatives, associated with the O-H bond stretching (3340 cm^−1^), C-H bond stretching of the methyl and methylene groups (2935 and 2880 cm^−1^), as well as C=C bond stretching of the aromatic ring (1600, 1510 and 1420 cm^−1^). Other bands visible in the ligS spectrum are connected with asymmetric and symmetric bending of the C-H bond in the methyl group (1450 cm^−1^), scissoring vibrations of the C-H bond in the methylene group (1370 cm^−1^), and stretching of the C-O bond in phenols (1340 cm^−1^). Bands associated with the asymmetric and symmetric stretching of the -SO_2_- group at 1200 and 1160 cm^−1^ and the asymmetric stretching of ether C-O-C linkage at 1137 cm^−1^ overlap, forming the next band visible on the spectrum. Primary alcohol C-O bond stretching results in a single band at 1029 cm^−1^. The band located at 652 cm^−1^ is formed as a result of the sulfonyl group’s S-O bond vibrations and is considered a band typical for lignosulfonates [77,78]. The spectrum acquired for the lignosulfonic acid (ligH) sample does not differ significantly from the ligS spectrum.

To observe the effect of modification with a chlorinating agent, the bands of interest are those connected with the sulfonyl group. All of the materials which underwent reaction with a chlorinating agent display bands associated with asymmetric and symmetric stretching in the -SO_2_- group, present at 1200, 1140, and 1020 cm^−1^ and the observed intensity is significantly more pronounced than for the reference samples. Additionally, spectra obtained for the materials modified with PCl_5_ display the occurrence of two new bands: one at 980 cm^−1^, which partially overlaps with the sulfonyl band at 1020 cm^−1^, and the other at 505 cm^−1^. Those signals result from the presence of phosphorus trichloride (PCl_3_) [79], a by-product of the employed chlorinating agent.

After the second step of lignin materials modification with dihexylamine (Figure 2b), the absorption bands located between 3000 and 2800 cm^−1^, which are attributed to the vibrations of the C-H bonds present in the methyl and methylene groups, see a substantial increase in intensity. Those bands originate from asymmetric and symmetric stretching of the aforementioned bonds in methyl (2960, 2870 cm^−1^) and methylene groups (2935, 2850 cm^−1^). The observed increase corresponds to the introduction of dihexylamine since each molecule contains two hexyl chains. As for the sulfonamides, the characteristic bands include weak bands present at 1380–1320 cm^−1^, which are attributed to the asymmetric stretching of the -SO_2_- group, as well as bands in the range of 1200–1140 cm^−1^, which are connected with the symmetric stretching. Vibrations stemming from the stretching of the N-S bond cause the appearance of a weak band in the 950–860 cm^−1^ range.

### 2.3. Solid-State Nuclear Magnetic Resonance (SS-NMR)

**Figure 3:**
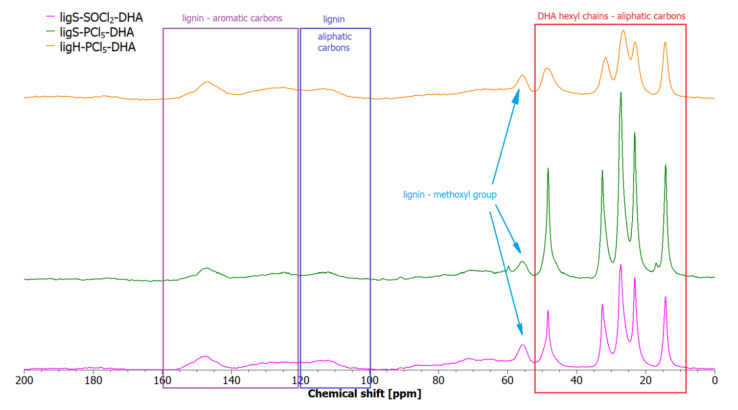
Solid-state ^13^C NMR spectra of the lignin materials after the second step of modification.

The chemical structure of the modified lignin samples was further investigated by the means of ^13^C SS-NMR spectroscopy, and the obtained spectra are presented in Figure 3. Spectra for all the samples exhibit a uniform pattern, with only subtle differences between each. Between 14.4 and 50 ppm, the observed peaks originate from the carbon atoms in hexyl chains of dihexylamine. The peak at 14.4 ppm chemical shift corresponds to the carbon atoms farthest from the nitrogen, whereas the peak at 49 ppm, to the carbons closest to the nitrogen. The peaks located at 23, 26.6, and 31.8 ppm are associated with the middle carbons. Since the hexyl chains contain six carbon atoms, there should be another peak between 20 and 35 ppm, but it is not visible due to the overlapping of other peaks. Methoxyl groups present in the lignin structure give a signal at 56 ppm. Between 100 and 160 ppm, it is difficult to distinguish between signals due to heavy overlapping, although a broad peak between 100 and 120 ppm consists of the signals related to the aliphatic carbons, whereas between 120 and 160 signals are related to the aromatic carbon atoms. The only distinctive signal is observable at 147 ppm and can be attributed to the quaternary carbon atoms present in sinapyl or guaiacyl units [80].

Recent years have brought significant progress in NMR analysis of native lignins, yet there are only a few reports dealing with the NMR study of lignosulfonates [81]. One of the approaches on the topic, a ^1^H and ^13^C NMR research study on 15 model compounds representing various structures of the lignosulfonate building blocks, was proposed by Lutnaes et al. [82]. The results reported by our group are in line with their findings.

### 2.4. Wide-Angle X-ray Diffraction (WAXD)

**Figure 4:**
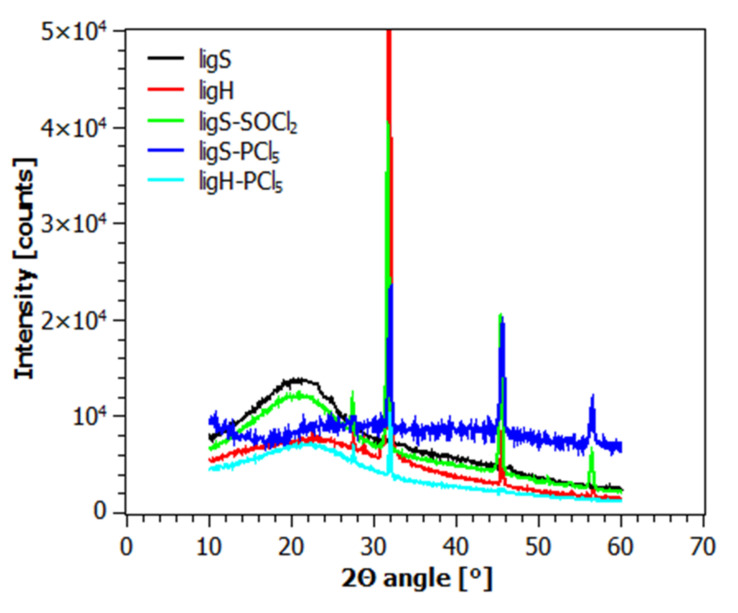
Diffraction patterns of the materials after the first step of modification.

**Figure 5:**
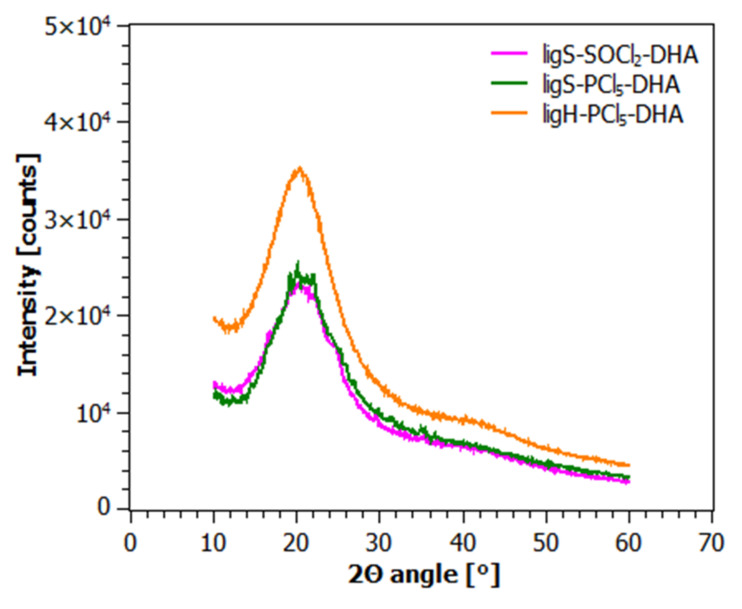
Diffraction patterns of the materials after the second step of modification.

Figure 4 and Figure 5 present diffractograms acquired for the investigated samples after the first (Figure 4) and the second step of modification (Figure 5).

Diffraction patterns of ligH and the materials modified with chlorinating agents display two crystalline reflexes, at 32° and 45.5° of the 2Θ angle. The appearance of these reflexes can be explained by the presence of sodium chloride in the samples [83], a by-product of the hydrolysis reaction (ligH) as well as chlorinating reaction (ligS-SOCl_2_, ligS-PCl_5_, ligH-PCl_5_). However, this is not the case for the samples investigated after the second step of modification (Figure 5). As one can observe in Figure 4 and Figure 5, all of the investigated materials exhibit a broad, amorphous halo in the range of 7 to 30° of the 2Θ angle, which confirms the amorphous structure of sodium lignosulfonate and its derivatives.

### 2.5. Scanning Electron Microscopy (SEM)

**Figure 6:**
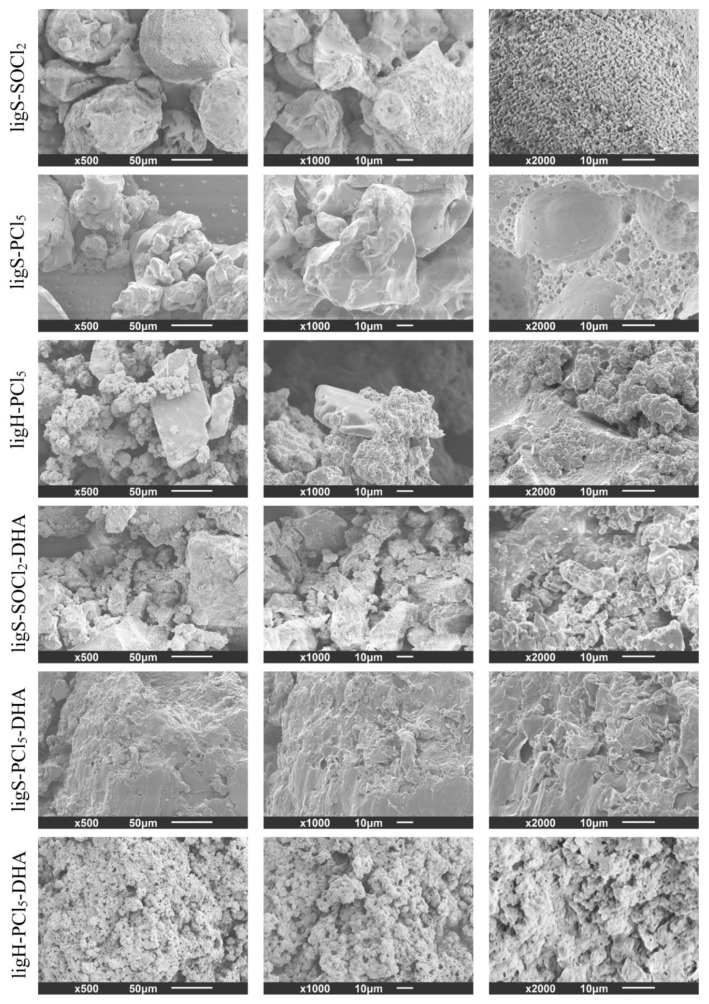
Morphology of lignin derivatives obtained after each step of the modification procedure.

To assess the morphology of the obtained materials, SEM imaging of the samples’ surfaces was employed (Figure 6). The ligS-SOCl_2_ material exhibited an irregular structure, with grains composed of crystal-like particles and with sporadically occurring pores. The structure observed for the material after the second stage of modification (ligS-SOCl_2_-DHA) was slightly different, without as many crystal-like particles contributing to the characteristic appearance of the aforementioned sample. In the case of the ligS-PCl_5_ sample, the observed grains were bigger and with a much smoother surface, although higher magnification revealed the existence of highly porous areas. Such high porosity was not maintained after the second step of the modification, and the surface of the grains became more irregular and uneven, along with the formation of platelet-like structures on the exterior. The ligH-PCl_5_ retained the cauliflower-like structure of the lignosulfonic acid, with occasionally occurring regularly shaped structures with smooth surfaces, which may be locally crystallized sodium chloride. Higher magnifications also indicate the porosity of the material. In this case, morphological changes after the modification with dihexylamine are only minute, since the ligH-PCl_5_-DHA sample displays the cauliflower-like structures as well.

### 2.6. Thermogravimetric Analysis (TGA)

**Figure 7:**
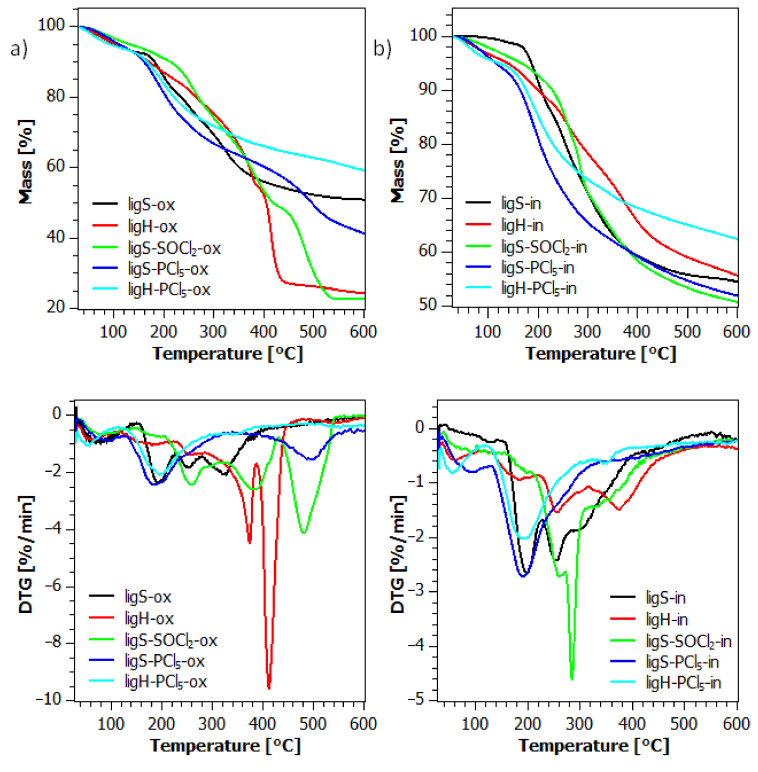
TG and DTG curves of the materials after the first step of modification during thermo-oxidative (**a**) and pyrolytic (**b**) degradation.

**Figure 8:**
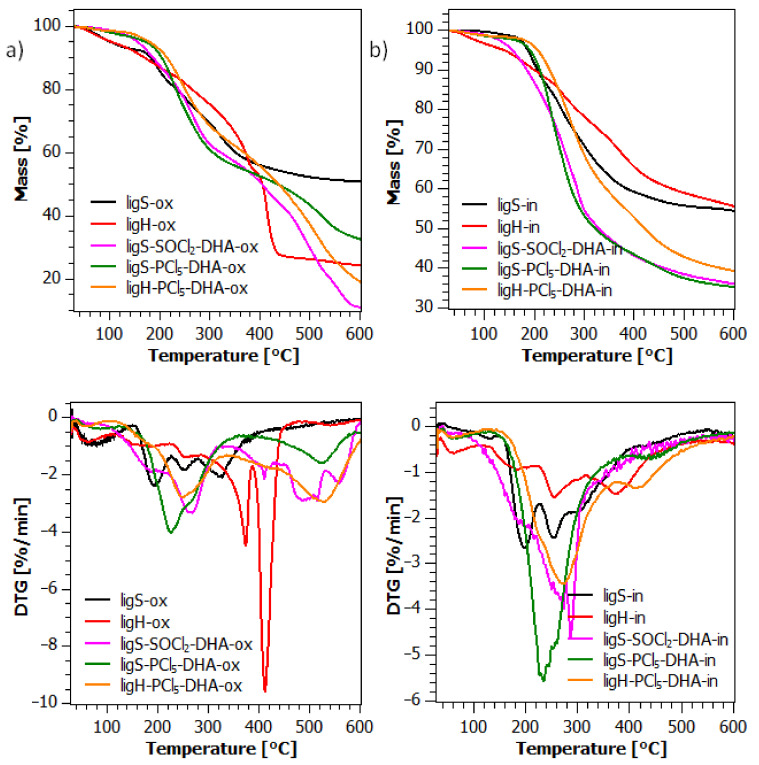
TG and DTG curves of the materials after the second step of modification during thermo-oxidative (**a**) and pyrolytic (**b**) degradation.

Thermogravimetric analysis was performed in the temperature range from 30 to 600 °C. Figure 7 presents TG and DTG curves obtained in both synthetic air and nitrogen atmosphere, both for reference samples and for the lignosulfonyl chlorides, whereas Figure 8 presents the results obtained for the sulfonamide lignin derivatives.

TG profiles presented in Figure 7a were obtained under thermo-oxidative conditions for reference samples and materials after the first stage of modification. Although all of the samples were dried prior to the measurements, the investigated materials exhibit about 5–10% weight loss below 200 °C, which is probably related to the evaporation of the residual moisture. As shown on the DTG profile, ligS and ligH display four stages of thermo-oxidative degradation, ligS-SOCl_2_ shows five stages, whereas ligS-PCl_5_ and ligH-PCl_5_ have three stages. The degradation behavior of the obtained lignosulfonyl chlorides differs depending on the used chlorinating agent. Up to ca. 200 °C, materials that underwent chlorination with PCl_5_ display a degradation pattern very similar to the raw material, with the initial mass loss below 100 °C and with a similar rate of decomposition. After the maximum mass loss (183 °C for ligS-PCl_5_ and 156 °C for ligH-PCl_5_), the rate of thermal decomposition decreases and remains at a similar level until the end of the analysis. In the case of the ligS-SOCl_2_ sample, its degradation starts slower, with the two first stages occurring at similar temperatures to materials modified with PCl_5_. Then, another three stages of thermal degradation occur with an increasing rate of decomposition, which peaks at 481 °C (T_max_) in the fifth stage. This sample also shows the least amount of char at 600 °C, which may point toward the destruction of the polymeric network of lignin, allowing for a more thorough burning of the material. Aside from ligS-SOCl_2_, which displays elevated temperatures of T_5%_, T_20%,_ and T_max_, materials after the first step of modification demonstrate inferior thermo-oxidative resilience. This is especially pronounced for the ligH-PCl_5_ sample, in which 5% mass loss temperature (T_5%_) is below 100 °C.

The results obtained under inert (nitrogen) conditions, presented in Figure 7b, indicate that materials after the first step of modification, as well as hydrolyzed lignosulfonate (ligH), are characterized by inferior thermal properties than the raw material (ligS). The degradation of obtained lignosulfonyl chlorides under inert conditions displays similar trends to those present in the thermo-oxidative degradation. Both ligS-PCl_5_ and ligH-PCl_5_ exhibit two first steps of decomposition at temperatures comparable with ligS, with T_max_ below 200 °C. Subsequently, the rate of pyrolytic degradation decreases with the increasing temperature. For the sample chlorinated with SOCl_2_, the rate of thermal degradation gradually increases, with T_max_ occurring at 285 °C. Analogous to that under thermo-oxidative conditions, the amount of char at 600 °C is the smallest among the samples investigated in this part of the analysis.

After the modification with DHA (Figure 8a), the obtained materials manifest a three-step thermal degradation pattern. Values of the T_5%_ and T_20%_ temperatures are increased in comparison to the raw material, although temperatures of the maximum mass loss (T_max_) are decreased, as shown in Table 2. Materials after the second step of modification, regardless of the chlorinating agent used in the previous step, display a more uniform degradation behavior under the thermo-oxidative conditions. The first stage of decomposition occurs under 100 °C. Then the second stage of degradation occurs, characterized by the maximum mass loss, with T_max_ ranging from 220 to 270 °C, however, the ligS-SOCl_2_-DHA sample starts degrading earlier and at a slower rate than ligS-PCl_5_-DHA and ligH-PCl_5_-DHA. The third stage takes place above 500 °C, with the rate of degradation reaching its peak around 530 °C. It is also worth mentioning that the residue at 600 °C for all of the modified materials is significantly lower than for the raw material, especially for the ligS-SOCl_2_-DHA material. Such behavior may be caused by the change in the carbon composition of the materials—the structure of lignins is heavily based on the aromatic building units, with a relatively small quantity of the aliphatic carbons, thus making the unmodified lignosulfonate more prone to undergo an incomplete degradation and formation the charred layer on the surface [84,85]. By the introduction of dihexylamine, a molecule with two hexyl chains, the ratio of aliphatic carbons is shifted, promoting a more uniform decomposition of the materials.

For the materials after the second step of the modification, investigated under the inert atmosphere, there is an increase of the T_5%_ temperature in the case of materials, which underwent chlorination with PCl_5_. However, it is not the case for the ligS-SOCl_2_-DHA sample, which displays a lower temperature of the 5% weight loss than the reference material. Both ligS-PCl_5_-DHA and ligH-PCl_5_-DHA display a sharp increase in degradation rate above 200 °C, whereas the ligS-SOCl_2_-DHA starts degrading earlier and at a slower rate. All of the modified materials display higher T_max_ temperature than the raw material. In the pyrolytic degradation, the investigated samples show an increase in the char residue at 600 °C in comparison with the measurements carried out under the thermo-oxidative conditions; however, the phenomenon of sulfonamide lignin derivatives leaving less charred residues after degradation remains true also for the non-oxidative circumstances.

Although it is difficult to consider the obtained lignosulfonyl chlorides as more thermally stable than the raw material, since their thermal properties differ in accordance with the employed method of synthesis, there is an improvement in some thermal parameters after the second step of modification, such as T_5%_ and T_max_. Such observation may be explained by the formation of sulfonamide. It is confirmed for cellulose nanocrystals obtained via acidic hydrolysis using different acids, that those formed as a product of hydrolysis with sulfuric acid have the least favorable thermal stability [86]. During the hydrolysis, hydroxyl groups on the surface of the nanocrystals may undergo a side reaction of esterification, which results in the formation of the sulfonyl groups and even their small number causes a visible deterioration in the thermal properties of cellulose [87]. The formation of sulfonamide from the sulfonyl group may inhibit desulfonation and shift it toward higher temperatures, resulting in enhanced thermal properties of the obtained sulfonamide derivatives of lignin. Similar observations were made in our previous work, in which we investigated lignosulfonamides obtained using chlorosulfonic acid [71].

While the results do not indicate an overall enhancement of the thermal stability of the obtained modified lignins, the improvement of their decomposition parameters up to ca. 230 °C is apparent. DHA-modified lignosulfonates were prepared as prospective fillers for the composites with a polyhydroxybutyrate (PHB) matrix. PHB is usually processed at temperatures between 160 and 220 °C, so in the processing window, obtained lignin sulfonamide derivatives are characterized by better thermal stability than the raw material. For this reason, the thermal parameters of the modified lignosulfonates are satisfactory from the viewpoint of future applications as polyhydroxyalkanoates modifiers.

## 3. Materials and Methods

### 3.1. Materials

Sodium lignosulfonate (STARLIG^®®^Na98S, industrial grade) in the form of yellow powder was supplied by Lignostar International BV (Hague, the Netherlands). Chloroform (analytical grade), thionyl chloride (reagent grade), phosphorus pentachloride (reagent grade), sodium hydroxide (analytical grade), and dihexylamine (97%) were purchased from Sigma-Aldrich (Darmstadt, Germany). Hydrochloric acid of analytical purity (35–38 wt%) was purchased from Chempur (Piekary Śląskie, Poland). All of the chemicals were used as received.

### 3.2. Chemical Modification of Sodium Lignosulfonate

#### 3.2.1. Modification with Thionyl Chloride (Synthesis Route A)

An amount of 47 g of sodium lignosulfonate (ligS) dried at 90 °C for 24 h under vacuum conditions was placed in a round bottom flask equipped with a condenser, followed by the addition of 940 mL of chloroform (CHCl_3_). Then, 16 g/9.8 mL of thionyl chloride (SOCl_2_) was added in small portions to the mixture. The contents of the flask were then heated to 50 °C and the reaction was carried out for 5 h. After cooling down to room temperature, the mixture was concentrated using a rotary evaporator at 50 °C with a 50 rpm rotating speed and under 500 mbar pressure. The obtained suspension containing lignosulfonyl chloride (ligS-SOCl_2_) was then dried at 60 °C until constant mass.

#### 3.2.2. Modification with Phosphorus Pentachloride (Synthesis Route B)

An amount of 54 g of ligS dried under the same conditions as above and 30 g of phosphorus pentachloride (PCl_5_) dissolved in 300 mL of chloroform were introduced into a round-bottom flask and mixed. After the flask was equipped with a condenser, another 300 mL of CH_3_Cl was added to the flask. Subsequently, the reagents were heated to 65 °C and reacted for 16 h. After the reaction, the resultant suspension was cooled down and then concentrated in a rotary evaporator at 50 °C, 50 rpm, and 700 mbar. The remaining thick suspension was dried in an oven at 60 °C to obtain dry lignosulfonyl chloride (ligS-PCl_5_).

#### 3.2.3. Modification with Phosphorus Pentachloride with Additional Acidolysis (Synthesis Route C)

This method employed the acidolysis of sodium lignosulfonate in hydrochloric acid (HCl), as described in our previous paper [71]. 200 g of ligS dried under as-above conditions was used to obtain powdered lignosulfonic acid (ligH), which was later reacted with phosphorus pentachloride, yielding lignosulfonyl chloride (ligH-PCl5). 34 g of ligH and 29 g of PCl_5_ were placed in a round-bottom flask and mixed. Then, the vessel was equipped with a condenser and 400 mL of CH_3_Cl was added to the mixture. After reaching 65 °C, the reaction was continued for another 16 h. After cooling down to room temperature, the mixture was placed in a rotary evaporator and concentrated until the desired degree under 50 °C, 50 rpm rotating speed and 700 mbar conditions. Then, the concentrated suspension was dried at 60 °C until constant mass to yield lignosulfonyl chloride (ligH-PCl_5_).

#### 3.2.4. Second Step of Modification

All of the obtained lignosulfonyl chlorides were subjected to the second step of the employed modification procedure, i.e., reaction with the dihexylamine, as it was described in our previous work [71]. In three flasks, NaOH solution, dihexylamine, and the corresponding lignosulfonyl chlorides were reacted. After cooling the reaction mixtures down to room temperature, a solid product was formed in the case of lignins modified with PCl_5_. The residue was filtered and washed thoroughly with distilled water and dried in ambient conditions, followed by another filtration with hot water. As for the material chlorinated in the previous step using SOCl_2_, there was no visible solid product and the contents of the flask were poured into a paper funnel. After separation of the yellow-colored filtrate, on the paper funnel remained a brown slurry, which solidified after two weeks. The solid product was dried for a few days in ambient conditions, then washed thoroughly with hot distilled water. All of the obtained samples were dried in a vacuum oven at 90 °C for 24 h before measurements.

### 3.3. FTIR Spectroscopy

Fourier transform infrared (FTIR) spectra were collected using a Nicolet iS5 spectrometer (Thermo Fisher Scientific, Waltham, MA, USA) equipped with a diamond attenuated total reflection (ATR iD7) probe, in the wavenumber range of 4000–400 cm^−1^.

### 3.4. Solid-State Nuclear Magnetic Resonance

Due to the insolubility of the investigated samples in the typical solvents used in NMR analysis, the chemical structure of the materials was assessed using ^13^C Solid-State Nuclear Magnetic Resonance. The spectra were recorded on NMR Bruker Avance III 500 MHz spectrometer (Bruker Corporation, Billerica, MA, USA). The instrument was equipped with a 4 mm probe. The number of scans for each sample was 512. The measurements were performed at a rotation frequency of 8 kHz with resonance frequencies of 128.5 and 500.1 MHz, with a 2 ms contact time. The chemical shifts for each sample were calculated from tetramethylsilane (TMS).

### 3.5. WAXD

Bruker D2 Phaser diffractometer (Bruker Corporation, Billerica, MA, USA) equipped with a Cu lamp (λ = 1.542 Å) was used to acquire diffractograms of the obtained materials at room temperature. Diffraction patterns were recorded in the 2Θ range of 3–60° with an increment of 0.1°.

### 3.6. SEM

The surface morphology of the obtained materials was investigated by a JEOL JSM-6010LA Analytical Scanning Electron Microscope (JEOL Ltd., Tokyo, Japan). The images were acquired at an accelerating voltage of 5 kV and 10 mm working distance. Before the investigation, each sample was coated with a 4 nm thick layer of gold.

### 3.7. TGA Analysis

Thermogravimetric analysis was performed using Netzsch TG 209F1 Libra analyzer (Netzsch GmbH, Selb, Germany). During the measurements, samples were placed in corundum crucibles. Thermogravimetry was carried out in a temperature range of 30–600 °C, at a heating rate of 10 K/min under oxidative (synthetic air) and inert (nitrogen) atmosphere.

## 4. Conclusions

In this work, sodium lignosulfonate was subjected to a two-step modification procedure: the first stage employed different chlorinating agents—SOCl_2_ and PCl_5_—to obtain lignosulfonyl chlorides, which were reacted in the second stage with dihexylamine to yield sulfonamide derivatives of lignin. Results of the FTIR spectroscopy indicate the existence of alkyl chains in the modified materials and confirm the bonding between sulfur and nitrogen in the sulfonamide group. ^13^C SS-NMR assessment provided information on the carbon composition of the sample, confirming the incorporation of dihexylamine into the structure of lignosulfonates and the subsequent formation of the sulfonamide. WAXD analysis has shown that no changes in the internal structure occur during the modification, with only slight morphological changes on the surface, confirmed by SEM microscopy. Depending on the employed route of modification, the materials displayed differences in the mechanism of thermal decomposition. After the first step of modification, for the lignosulfonates modified with SOCl_2_ (route A) the thermal degradation started at lower temperatures, propagated at a slower rate, and reached the maximum mass loss at higher temperatures. Samples modified with PCl_5_ (routes B and C) displayed thermal decomposition similar to the raw material, reaching the maximum mass loss below 200 °C. The T_5%_ temperature of the obtained lignosulfonyl chlorides was enhanced in comparison with the raw materials. After the second step of the modification procedure, materials obtained via the reaction with dihexylamine exhibit an increase in the value of T_5%_ and T_20%_ temperatures. This phenomenon may be related to the sulfonamide group acting as an inhibitor to desulfonation and shifting it towards higher temperatures, improving the thermal degradation parameters. Char residue left after the thermogravimetry of the modified lignins has a lower mass than in the case of the raw material, which may be caused by the introduction of alkyl chains from dihexylamine, thus shifting the ratio of aromatic to aliphatic carbons and influencing the mechanism of thermal degradation. The results show an improvement in thermal properties of DHA-modified lignins in comparison with the raw material up to 230 °C, which is adequate for their planned application in composites with polyhydroxybutyrate matrix. The described two-step method of chemical modification of lignosulfonates, involving both chlorination and formation of lignin sulfonamide derivatives by the reaction with secondary amines, may be potentially used to obtain functional, value-added lignin-based materials, which can be used as a filler in biopolymer matrices. Currently, our team is focused on obtaining other sulfonamide derivatives, using different types of secondary amines, and determining how their properties change in accordance with the structure of the amine used.

## Figures and Tables

**Table 1:** Description of the materials investigated in the paper.

Material	Description
ligS	Pure sodium lignosulfonate
ligH	Lignosulfonic acid, obtained via reaction of ligS with hydrochloric acid (HCl)
ligS-SOCl_2_	Lignosulfonyl chloride, a product of the reaction of ligS with SOCl_2_
ligS-PCl_5_	Lignosulfonyl chloride, a product of the reaction of ligS with PCl_5_
ligH-PCl_5_	Lignosulfonyl chloride, a product of the reaction of ligH with PCl_5_
ligS-SOCl_2_-DHA	Main products, sulfonamide derivatives of lignin, formed in the reaction of the corresponding lignosulfonyl chloride with dihexylamine
ligS-PCl_5_-DHA	
ligH-PCl_5_-DHA	

**Table 2:** Parameters of the thermal decomposition of the lignin-based materials.

Title 1	Degradation in Synthetic Air				Degradation in Nitrogen			
	T_5%_ [°C]	T_20%_ [°C]	T_max_ [°C]	Char at 600 °C [%]	T_5%_ [°C]	T_20%_ [°C]	T_max_ [°C]	Char at 600 °C [%]
ligS	102	234	327	50.9	187	255	199	54.5
ligH	104	264	412	24.4	139	285	374	55.7
ligS-SOCl_2_	131	268	481	22.7	167	273	285	50.8
ligS-PCl_5_	110	205	183	41.3	115	204	190	51.9
ligH-PCl_5_	91	220	156	59.2	121	230	188	62.5
ligS-SOCl_2_-DHA	155	237	268	11.0	156	229	287	36.0
ligS-PCl_5_-DHA	172	232	226	32.6	191	232	235	35.4
ligH-PCl_5_-DHA	179	250	249	19.1	207	265	274	39.4

## Data Availability

The data are contained within the article.
